# Post-Acute Sequelae of SARS-CoV-2 and Kidney Events in U.S. Active Component Service Members, March 1, 2020–September 30, 2022

**Published:** 2024-12-20

**Authors:** Kayli Hiban, Sithembile L. Mabila, Jessica H. Murray, Alexis A. McQuistan, Natalie Y. Wells

**Affiliations:** 1 Epidemiology and Analysis Branch, Armed Forces Health Surveillance Division, Public Health Directorate, Defense Health Agency, Silver Spring, MD

## Abstract

Early evidence suggests that COVID-19 is linked to kidney-related events in older and hospitalized patients. This link has not, however, been explored among a younger, healthy population such as U.S. active component service members (ACSMs). This retrospective cohort study assessed the incidence of post-acute sequelae of SARS-CoV-2 (PASC) and kidney events between March 1, 2020 and September 30, 2022 in ACSMs with no prior history of kidney events. Among the study population (n=831,780), 1,975 (0.2%) kidney events were reported between 31 days and 6 months after COVID-19 test dates. The incidence rate of any kidney event was slightly higher among ACSMs who tested negative for COVID-19 (10.6 per 10,000 person-years) compared to ACSMs who tested positive (9.8 per 10,000 person-years). In adjusted models of incidence rate ratios (IRRs), older ACSMs evinced significantly higher rates of kidney events compared to younger ACSMs, and COVID-19 vaccination had a protective effect; this was true in both the COVID-19-positive and -negative groups, although the IRR magnitude was stronger in the COVID-19-positive group. PASC did not lead to an increased incidence of kidney events compared to the COVID-19-negative group among ACSMs, an overall young and healthy population.

**What are the new findings?:**

PASC-related kidney events were rare among a young, healthy population of ACSMs without prior history of kidney events. The incidence of kidney events among ACSMs was not higher in the COVID-positive group compared to the group that tested negative for COVID.

**What is the impact on readiness and force health protection?:**

The detection and management of PASC in ACSMs is essential to prevent morbidity and ensure optimal health of the force, particularly among ACSMs of older age and at higher risk of PASC-related outcomes.

## 1 BACKGROUND

Severe acute respiratory syndrome coronavirus-2 (SARS-CoV-2), the virus that causes coronavirus disease 2019 (COVID-19), infected nearly 190,000 U.S. service members between January 1, 2020 and August 31, 2021. Of those cases, 1,760 resulted in hospitalizations and 45 resulted in deaths.^1^ While individuals who are older or have a comorbidity are more at risk for severe illness from the virus,^2,3,4^ COVID-19 also poses health risks to young and healthy individuals, including active component service members (ACSMs).

The most common health consequences of COVID-19 occur in the acute illness stage, including influenza-like symptoms, loss of taste or smell, nausea, and diarrhea.^5^ Growing evidence suggests, however, that COVID-19 is a multi-organ disease, sometimes causing persistent symptoms after recovery. The U.S. Centers for Disease Control and Prevention defines long COVID as new, returning, or ongoing health problems occurring at least 4 weeks after COVID-19 infection.^6^ Long COVID has also been referred to as long haul COVID, post-COVID-19 conditions, chronic COVID, or post-acute sequelae of SARS-CoV-2 (PASC). Each of these terms is associated with a specific timeline for persistence of symptoms after COVID-19 infection; for example, PASC refers to the onset or persistence of symptoms more than 4 weeks after infection, and long haul COVID refers to symptom persistence more than 100 days after infection.^7^ For the purposes of this study, PASC refers to long COVID. PASC can adversely affect a variety of organ systems, including the respiratory, cardiovascular, neurological, and genitourinary systems, regardless of severity of COVID-19 illness.^8^

Early evidence suggests that COVID-19 is linked to kidney-related events, leading to more severe disease and increased hospital mortality.^9^ In the acute stage of infection, nearly one-third of patients hospitalized with COVID-19 were diagnosed with acute kidney injury (AKI).^10^ Patients hospitalized for COVID-19 more frequently experienced AKI, hematuria, and proteinuria and were less likely to have kidney function recovery than those hospitalized for influenza.^11,12^ Similarly, kidney diseases are common following COVID-19 recovery, even among patients without kidney damage in the acute stage. Several studies of U.S. military veterans showed a significantly increased risk and burden of kidney diseases among 30-day COVID-19 survivors; this risk increased with severity of COVID-19 infection, but was present even for mild cases.^13,14,15^

Evidence for the association between PASC and kidney events is growing, but current research has focused largely on hospitalized patients and older populations. To evaluate its full burden, it is important to study PASC kidney events among a younger population such as ACSMs. The objectives of this study were to determine the incidence and incidence rate ratio (IRR) of selected kidney events in ACSMs occurring 31 days to 6 months after a COVID-19 test date, and by COVID-19 test status.

## 2 METHODS

This retrospective cohort study assessed PASC and kidney events among ACSMs from March 1, 2020 through September 30, 2022. Incidence of selected kidney events among those who tested positive for COVID-19 was determined through reverse transcription-polymerase chain reaction (RT-PCR) in comparison to those who tested negative. A total of 888,588 ACSMs with PCR COVID-19 tests were identified from March 1, 2020 until March 31, 2022. COVID-19-positive individuals were identified by positive PCR COVID-19 tests, and COVID-19-negative individuals were identified by negative PCR COVID-19 tests. Individuals without a PCR test but who were tested with antigen or unknown test types were not included in either group. Only service members who never tested positive by PCR for COVID-19 during the surveillance period were included in the COVID-19-negative group. Individuals with only negative PCR test results but positive or suspect results to antigen or unknown test types (n=38,712) were excluded from the negative group (**Figure**). The first negative test result was used as the incident date for the COVID-19-negative group. ACSMs with multiple COVID-19-positive tests (n=1,008) during the study were excluded, as it was not possible to ascertain if a kidney event was due to PASC or a subsequent COVID-19 infection during the acute stage (**Figure**). ACSMs with histories of kidney events prior to a COVID-19 test (n=17,086) were excluded from the study, but those who experienced kidney events within 30 days after their COVID-19 tests were not excluded. The final cohort comprised 104,422 ACSMs with 1 positive PCR test and 727,358 with only negative PCR tests during the surveillance period (**Figure**).

The Defense Medical Surveillance System (DMSS) was utilized to capture selected kidney-related encounters with International Classification of Diseases, 10th Revision (ICD-10) codes, for AKI, chronic kidney disease, proteinuria, nephrosis, nephritis, renal sclerosis, or other kidney events, in the first or second diagnostic position (**Table 1**) through September 30, 2022, to allow 6 months of follow-up. Six months of follow-up was necessary because PASC-related symptoms can persist 24 weeks or more after COVID-19 infection.^7,16^ Kidney events within 30 days following a COVID-19 test were considered complications of acute COVID-19 infection, while kidney events more than 30 days, until 6 months, after a COVID-19 test date were considered PASC. Similar methods were used to evaluate the PASC burden among U.S. veterans.^15^ An individual was counted once per kidney event type within the follow-up period. Encounters with kidney-related ICD-9/10 codes in the first or second diagnostic position were also used to determine prior history of kidney events for exclusion. For COVID-19 vaccination status, fully vaccinated was defined as the completion of a vaccination series at least 14 days prior to COVID-19 testing.

Person-time contributions for each ACSM were obtained for March 1, 2020 until September 30, 2022. Person-time was censored when a service member left the active component, at the conclusion of 6-month follow-up, or at the end of the surveillance period, whichever occurred first. Crude and multivariable Poisson regression models (adjusted for sex, age category, race and ethnicity, military service, and COVID-19 vaccination status) assessed the IRR of kidney events per 10,000 person-years (p-yrs), from 31 days to 6 months following COVID-19 testing, by test status.

## 3 RESULTS

There were 104,422 ACSMs who tested positive for COVID-19 and 727,358 who tested negative among the study population (**Figure**). The average age at COVID-19 test date was 27 years. There were 694 (0.7%) COVID-19 hospitalizations reported among those who tested positive. There were 1,975 kidney events reported between 31 days and 6 months after COVID-19 test date among the cohort: 244 (0.2%) in the COVID-19-positive group and 1,731 (0.2%) in the COVID-19-negative group. The proportions of each of the kidney events were relatively similar among the COVID-19-negative and the COVID-19-positive group (data not shown). Kidney-related hospitalizations were very low (n=87) in this study, with 11 among the COVID-positive group and 76 among the negative group. Overall, the incidence rate of PASC-related kidney events was lower among ACSMs who tested positive for COVID-19 (9.8 per 10,000 p-yrs) compared to kidney events among the COVID-19-negative group (10.6 per 10,000 p-yrs) (**Table 2**). The 3 leading kidney events among both groups were ‘other’ kidney events, acute kidney injury, and proteinuria (**Table 2**). Among both groups, the incidence rate of kidney events increased with age and was highest among non-Hispanic Black ACSMs and Army service members (**Table 2**). The incidence rate of kidney events was higher among the COVID-19-positive group than the negative group among those 40 years and older and those unvaccinated prior to testing. PASC-related kidney events in the COVID-19-positive group were also higher among those unvaccinated prior to their COVID-19 test compared to those vaccinated (13.3 vs. 7.6 per 10,000 p-yrs) (**Table 2**). Moreover, the incidence rate of kidney events was higher among men compared to women (10.1 vs. 8.9 per 10,000 p-yrs) within the COVID-19-positive group, while the rate was similar for both sexes within the COVID-19-negative group (females: 10.8 per 10,000 p-yrs vs. males: 10.6 per 10,000 p-yrs) (**Table 2**).

Among both groups, the IRR of kidney events increased significantly with age in the older than 40 years age groups compared to the under-20-year age group. In the 40-44-year and 45-year and older groups, the magnitude of the IRR was higher in the COVID-positive group (aIRR 2.5 [95% CI, 1.3-4.8]; aIRR 3.4 [95% CI, 1.7-7.0]) compared to the negative group (aIRR 1.7 [95% CI, 1.3-2.1]; aIRR 2.5 [95% CI, 1.9-3.2]) (**Table 3**). COVID-19 vaccination had a significant protective effect against kidney events in both groups, although the effect was stronger among the COVID-positive group (aIRR 0.5; 95% CI, 0.4-0.7) compared to the negative group (aIRR 0.9; 95% CI, 0.8-1.0) (**Table 3**).

## 4 DISCUSSION

This study did not identify an overall increased incidence of kidney events in ACSMs who tested positive during the surveillance period for COVID-19 compared to those who tested negative. This finding is likely due to the demographic characteristics of the ACSM population in the study; COVID-19 hospitalizations were low (0.7%) meaning a majority of the COVID-19 cases were mild, and the average age (27 years) was low. Both of these factors have been linked with a lower risk of PASC in the existing literature. First, studies have shown that the risk of PASC-related kidney outcomes increases with the severity of acute COVID-19 infection and is lowest among non-hospitalized patients.^13,14,15^ Second, PASC is more common among older patients than younger patients.^17,18^

The results of the current study align with the findings of other studies on PASC and kidney events in older populations, in that the risk of kidney events increases with age. An analysis of Veterans Health Administration data showed a 47% increased risk of AKI (hazard ratio [HR] 1.5; 95% CI, 1.0-2.2) and a 15% increased risk of chronic kidney disease (HR 1.2; 95% CI, 1.1-1.3) among non-hospitalized 30-day COVID-19 survivors.^13^ In a similar study of veterans, PASC was associated with a 41% increased risk of AKI (HR 1.4; 95% CI, 1.3-1.5) and an 18% increased risk of chronic kidney disease (HR 1.2; 95% CI, 1.1-1.2). The burden of PASC-related AKI increased with older age.^15^ A third study of veterans found that PASC almost doubled risk of AKI in 30-day COVID-19 survivors (aHR 1.9; 95% CI, 1.9-2.0).^14^ Previous studies have focused on hospitalized patients^9,10,11,12^ and veterans with an average age of late 50s or 60s,^11,12,13,14,15^ while this population has an average age of 27 years and few hospitalizations. In this study, the rate of kidney events was similar among COVID-positive and -negative ACSMs for all age groups except for ages 40-44 years and 45 and older, where the rate was higher in the COVID-positive group. These findings add to the literature that PASC may increase the risk of kidney events in older populations, but in a younger population such as ACSMs without a history of kidney events, the overall incidence rate was not higher in COVID-positive individuals.

This study found a higher incidence of kidney events among non-Hispanic Black ACSMs, which is a disparity supported in the existing literature. In a study of U.S. veterans, the 6-month burden of PASC-related acute kidney injury was higher among Black patients compared to White patients,^15^ and a meta-analysis found that Black COVID-19 patients were at highest risk of AKI.^17^ Additionally, in this study COVID-19 vaccination had a protective effect on the incidence of PASC-related kidney events among COVID-positive ACSMs. COVID-19 vaccination has been linked to a significantly lower risk of PASC-related kidney diseases following COVID-19, which supports these findings.^19,20^

There were numerous limitations in this study. First, this study relied on PCR testing to determine COVID-19 test status. With the increasing use of at-home COVID-19 tests, ACSMs who tested positive may have been misclassified as COVID-negative, potentially understating the difference in incidence of PASC and kidney events compared to the COVID-19-negative group. The results were not adjusted for co-morbidities or medications, including nephrotoxic agents or medications prescribed during COVID-19 treatment, that may have affected ACSMs’ rates of kidney events. ACSMs are generally in good health, however, so the prevalence of disease and health conditions in this population tends to be low. Medical encounters for genitourinary diseases in 2021, for instance, accounted for only 2.8% of military member encounters overall.^21^ Additionally, this study found low hospitalizations for COVID-19 and kidney events. Moreover, this study did not utilize laboratory data to assess how many patients required kidney function follow-up after COVID-19 testing; intensity of follow-up for the COVID-positive and -negative groups is important in determining true incidence of PASC-related kidney events. Lastly, this study did not account for the dominant circulating COVID-19 variant, which affects risk of PASC, throughout the surveillance period. A study of veterans found that risk of PASC decreased over the course of the pandemic,^22^ which may have affected the findings of the current study. Future studies should account for increasing use of at-home COVID-19 tests, adjust for co-morbidities and medications, incorporate laboratory data, and consider COVID-19 variants.

Overall, kidney events were rare among U.S. ACSMs, and kidney event incidence was not higher among COVID-19-positive individuals compared those who were negative. The rate of kidney events increased significantly with age, however, which supports the findings of studies of older, hospitalized populations, in which PASC is associated with increased kidney event risk.

## Figures and Tables

**Table 1:** ICD-9 and ICD-10 Codes for Selected Kidney Events

Kidney Event	ICD-10^a^	ICD-9^a^
Acute kidney injury	N17.0, N17.1, N17.2, N17.8, N17.9	584.5, 584.6, 584.7, 584.8, 584.9, 586
Chronic kidney disease	I12.0, I12.9, I13.10, I13.11, I13.2, N18.1, N18.2, N18.3, N18.30, N18.31, N18.32, N18.4, N18.5, N18.9	403.01, 403.11, 403.91, 404.00, 404.10, 404.90, 404.02, 404.12, 404.92, 585*
Proteinuria	N06*, R80.0, R80.1, R80.8, R80.9	791, 593.6
Nephrosis, nephritis, and renal sclerosis	N00*, N01*, N03*, N04*, N05*, N14.4, N15.8, N15.9, N26.9	580.0, 580.4, 580.81, 580.89, 580.9, 582.0-582.2, 582.4, 582.81, 582.89, 582.9, 581.0-581.3, 581.81, 581.9, 583.0-583.2, 583.4, 583.6, 583.7, 583.81, 583.89, 583.9, 587
Other diseases of kidney	N13.0, N13.1, N13.2, N13.30, N13.39, N26.1, N28.0, N28.1, N28.81, N19	591, 588.1, 593, 593.71-593.73, 593.2, 593.1

Abbreviations: ICD-9, International Classification of Diseases, 9th Revision; ICD-10, International Classification of Diseases, 10th Revision; ICD-9-CM, International Classification of Diseases, 9th Revision, Clinical Modification; ICD-10-CM, International Classification of Diseases, 10th Revision, Clinical Modification.

^a^ICD codes for kidney events were chosen from the Clinical Classifications Software Refined (CCSR) for ICD-10-CM (and ICD-9-CM) Diagnoses (a family of databases developed as part of the Healthcare Cost and Utilization Project).

*Asterisk indicates that any ICD code that begins with the specified value is included.

**Figure:**
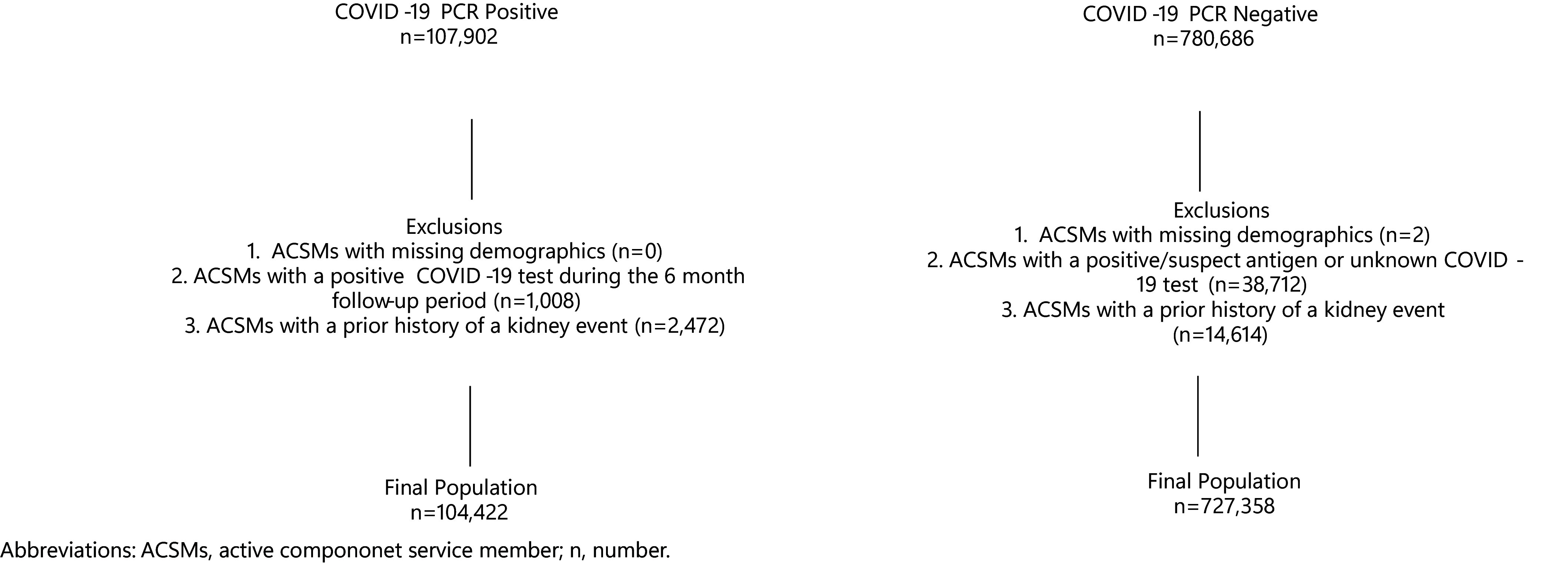
Study Population, March 1, 2020–March 31, 2022

**Table 2:** Incidence Rates of Kidney Events Among Active Component Service Members by COVID-19 PCR Test Result, March 1, 2020–September 30, 2022

	COVID-19-Positive			COVID-19-Negative		
	Individuals with a Kidney Event	Person-Time	Rate^a^	Individuals with a Kidney Event	Person-Time	Rate^a^
Overall	244	247,903.5	9.8	1,731	1,632,132.3	10.61
Sex						
Male	202	200,813.5	10.1	1,417	1,340,074.7	10.6
Female	42	47,090.1	8.9	314	292,057.6	10.8
Age group, y						
<20	13	14,622.0	8.9	120	117,367.8	10.2
20-24	69	85,431.2	8.1	482	554,343.3	8.7
25-29	43	60,726.9	7.1	336	379,318.8	8.9
30-34	39	40,137.0	9.7	252	253,048.5	10.0
35-39	35	28,276.6	12.4	254	182,905.7	13.9
40-44	27	12,629.0	21.4	151	90,829.6	16.6
>=45	18	6,080.9	29.6	136	54,318.7	25.0
Race and ethnicity						
White, non-Hispanic	116	125,680.7	9.2	885	890,533.5	10.0
Black, non-Hispanic	61	45,317.5	13.5	393	262,063.1	15.0
Hispanic	49	49,836.5	9.8	268	288,539.1	9.3
Other/unknown	18	27,068.9	6.7	185	190,996.6	9.7
Service branch						
Army	109	96,894.5	11.3	774	593,356.2	13.0
Navy	65	61,942.6	10.5	398	419,354.1	9.5
Air Force	56	54,758.4	10.2	359	385,078.6	9.3
Marine Corps	14	31,924.2	4.4	182	218,789.8	8.3
Coast Guard	0	2,383.9	0.0	18	15,553.6	11.6
Education						
High School or less	154	166,474.9	9.3	1,077	1,066,718.0	10.1
Some college	39	29,884.3	13.1	237	185,556.3	12.8
Bachelors or advanced degree	50	47,196.1	10.6	382	350,906.2	10.9
Other/unknown	1	4,348.2	2.3	35	28,951.8	12.1
Vaccination status prior to COVID-19 test						
Fully vaccinated	113	149,220.7	7.6	387	390,442.4	9.9
Not vaccinated	131	98,682.8	13.3	1,344	1,241,689.9	10.8
Kidney events^b^						
Acute kidney injury	56	247,903.5	2.3	457	1,632,132.3	2.8
Chronic kidney disease	24	247,903.5	1.0	189	1,632,132.3	1.2
Proteinuria	42	247,903.5	1.7	321	1,632,132.3	2.0
Nephrosis, nephritis, renal sclerosis	4	247,903.5	0.2	25	1,632,132.3	0.2
Other	118	247,903.5	4.8	739	1,632,132.3	4.5
Any kidney event	244	247,903.5	9.8	1,731	1,632,132.3	10.6

Abbreviations: COVID-19, coronavirus disease 2019; y, years.

^a^Rate per 10,000 person-years.

^b^Each kidney event counted once per person.

**Table 3:** Adjusted Incidence Rate Ratios for Any Kidney Event^a^ Among COVID-19 PCR Positive and COVID-19 PCR Negative Active Component Service Members, March 1, 2020–September 30, 2022

	COVID-19-Positive	COVID-19-Negative
	Adjusted IRR^b^ (95% CI)	Adjusted IRR^b^ (95% CI)
Sex		
Male	Reference	Reference
Female	0.9 (0.6-1.2)	1.0 (0.9-1.1)
Age group, y		
<20	Reference	Reference
20-24	0.9 (0.5-1.6)	0.9 (0.7-1.1)
25-29	0.8 (0.4-1.4)	0.9 (0.7-1.1)
30-34	1.1 (0.6-2.0)	1.0 (0.8-1.2)
35-39	1.4 (0.8-2.7)	1.4 (1.1-1.7)
40-44	2.5 (1.3-4.8)	1.7 (1.3-2.1)
>=45	3.4 (1.7-7.0)	2.5 (1.9-3.2)
Race and ethnicity		
White, non-Hispanic	Reference	Reference
Black, non-Hispanic	1.4 (1.1-2.0)	1.5 (1.3-1.7)
Hispanic	1.2 (0.8-1.6)	1.0 (0.9-1.1)
Other/unknown	0.7 (0.5-1.2)	1.0 (0.9-1.2)
Service branch		
Army	Reference	Reference
Navy	0.9 (0.7-1.2)	0.7 (0.7-0.8)
Air Force	1.0 (0.7-1.3)	0.8 (0.7-0.9)
Marine Corps	0.4 (0.2-0.7)	0.7 (0.6-0.9)
Coast Guard	0.0 (0.0-0.0)	0.9 (0.5-1.4)
Vaccination status prior to COVID-19 test						
Fully vaccinated	0.5 (0.4-0.7)	0.9 (0.8-1.0)
Not vaccinated	Reference	Reference

Abbreviations: COVID-19, coronavirus disease 2019; IRR, Incidence rate ratio; CI, confidence interval; y, years.

^a^Kidney events were counted from period of 31 days to 6 months following COVID-19 test date.

^b^Adjusted for sex, age, race and ethnicity, service, and COVID-19 vaccination status.

## AUTHORS' AFFILIATION

Epidemiology and Analysis Branch, Armed Forces Health Surveillance Division, Public Health Directorate, Defense Health Agency, Silver Spring, MD: Ms. Hiban, Dr. Mabila, Ms. Murray, Ms. McQuistan, Dr. Wells
